# Quality of Life in Patients with Focal Hyperhidrosis before and after Treatment with Botulinum Toxin A

**DOI:** 10.1155/2014/308650

**Published:** 2014-02-06

**Authors:** Anargyros Kouris, Kalliopi Armyra, Christos Christodoulou, Polixeni Karimali, Dimitrios Karypidis, George Kontochristopoulos

**Affiliations:** ^1^Department of Dermatology and Venereology, A. Sygros Hospital, 16121 Athens, Greece; ^2^2nd Department of Psychiatry, National and Kapodistrian University of Athens, Medical School, “Attikon” University Hospital, 12462 Athens, Greece; ^3^Department of Wound Healing, Cardiff University, Cardiff CF103EU, UK

## Abstract

The aim of this study is to assess the effectiveness of treatment with BTX-A in quality of life of patients suffering from primary focal hyperhidrosis. *Materials and Methods*. A total of 119 patients (62 females and 57 males) between 18 and 65 years suffering from moderate to severe focal hyperhidrosis were treated with BTX-A. Thirty-nine patients suffered from axillary hyperhidrosis, 47 patients from palmar hyperhidrosis, 12 patients from plantar hyperhidrosis, and 21 patients from palmar and plantar hyperhidrosis. A baseline and posttreated examination of patients 6 months after BTX-A is included. The Hyperhidrosis Disease Severity Scale (HDSS) was chosen to assess the disease severity and the modified Dermatology Life Quality Index was used (DLQI) to assess the quality of life. *Results*. Quality of life showed a significant improvement after treatment with BTX-A. The total DLQI score resulted significantly lower than the basal value (*P* < 0.0001). The seriousness of hyperhidrosis significantly decreased after the treatment (*P* < 0.0001). In addition, there was notable difference between the posttreatment DLQI scores and pretreatment severity of hyperhidrosis by sex. *Conclusions*. Treatment with BTX-A led to the reduction of disease severity and improvement of quality of life, while it is a safe, easy to use method with minimal side effects.

## 1. Introduction

Hyperhidrosis is a disorder characterized by excessive sweating, more than it is required for normal thermoregulation. Frequently, it interferes with patients' daily activities and may lead to emotional problems in their professional and social life. It is estimated that hyperhidrosis affects 3% of the US population [1]. It is divided into primary and secondary type. Primary hyperhidrosis is localized to the palms, soles, axillae, and face. It is caused by intense emotion or stress and it affects patients mainly during waking hours. A family history appears in 60–80% of affected individuals. Secondary hyperhidrosis can be localized or generalized and is associated with another systemic disorder such as genetic syndromes, malignancy, infection, endocrine, vasomotor, neurologic disorders, and drugs. The majority of hyperhidrotic patients have normal morphology of sweat glands, but there is an abnormal response to stimulation of sweat glands by hypothalamus [2].

The treatment of hyperhidrosis includes topical therapy, iontophoresis, oral medications (anticholinergic, a-adrenergic blockers), and surgical therapy. Recently, BTX-A has been used for the treatment of primary focal hyperhidrosis with excellent efficacy, safety, good tolerability and ease of use [2–8]. BTX-A belongs to a family of neurotoxins produced by the anaerobic bacillus *Clostridium botulinum*. BTX-A inhibits the release of acetylcholine from presynaptic cholinergic neurons. It has several applications in clinical medicine, such as the treatment of spasticity, blepharospasm, and strabismus [2, 9]. BTX-A has been approved by the Food and Drug Administration in the US for the treatment of axillary hyperhidrosis but it is also effective for palmoplantar disease. It is considered the treatment of choice when topical treatments are ineffective [2]. The effect lasts from 7 to 12 months for axillary hyperhidrosis with doses of 50 MU and from 4 to 6 months for palmar hyperhidrosis with doses of 120–200 MU [10]. Pain during injection and minor muscle weakness especially of the hands or feet are the major side effects of the treatment [10]. The efficacy of BTX-A can be measured by objective estimates such as gravimetry and Minor test, which assess the quantity of sweat production. Nevertheless the aforementioned methods are complex in clinical use and they do not evaluate the functional impairment and reduced quality of life of the patient due to hyperhidrosis [10]. Subjective estimates are the Hyperhidrosis Disease Severity Scale (HDSS), which measures disease severity and the Dermatology Life Quality Index (DLQI) which evaluates the impact of hyperhidrosis on daily activities. The use of these estimates is more indicative of the extent to which treatment helps the patient [10, 11]. The aim of the present study is to evaluate the impact on quality of life of BTX-A treatment in patients with primary focal hyperhidrosis measured by HDSS and DLQI.

## 2. Materials and Methods

From April 2011 to March 2012, patients referred to the 2nd Department of Dermatology and Venereology of Andreas Sygros Hospital, Athens, Greece, for treatment of moderate to severe focal palmar, plantar, and axillary hyperhidrosis who also showed impairment in their quality of life were enrolled in the study. Written consent was obtained from all patients. A total of 119 patients (62 females and 57 males) between 18 and 65 years were treated with BTX-A. Thirty-nine patients suffered from axillary hyperhidrosis, forty-seven patients suffered from palmar hyperhidrosis, twelve patients suffered from plantar hyperhidrosis, and twenty-one patients suffered from palmar and plantar hyperhidrosis. Pregnant or breast-feeding women and patients that were using systemic therapy that could interfere with neuromuscular activity were excluded from the study.

All patients had a complete evaluation of clinical assessment of the hyperhidrotic area. A baseline and posttreatment examination of patients 6 months after BTX-A was also included. The Hyperhidrosis Disease Severity Scale (HDSS) was chosen to assess the disease severity, according to the Canadian Hyperhidrosis Advisory Committee [11]. It is a single-item 4 point scale on which the patients rate the tolerability of their hyperhidrosis symptoms and the degree of interference with their activities that those symptoms are associated with. A score of 3 or 4 indicate severe hyperhidrosis, and a score of 1 or 2 indicate moderate hyperhidrosis. Moreover, the extension of hyperhidrotic area was assessed by the Minor-iodine starch test, in which an iodine solution was spread on the area of skin to be tested. After the solution dries, fine rich starch powder was applied. Sweat production then caused the mixture to turn dark blue, identifying the exact location of sweating in the target zone. The hyperhidrotic areas were marked using a demographic pen and each area was then subdivided into squares 1.5 × 1.5 cm. A dose of 0,10 mL of botulinum toxin type A (BOTOX; Allergan, Irvine, CA, USA) was injected intracutaneously into each area.

To assess the quality of life, a modified Dermatology Life Quality Index (DLQI) was used [10]. The questionnaire was modified from the standard version, changing the wording in question one from “symptomatic” to “sweaty,” to make it appropriate for application in hyperhidrosis. The DLQI is a validated 10-item questionnaire regarding leisure, personal relationships, daily activities, and treatment. The maximum score is 30, with 0 indicating the least impairment and 30 the most impairment in patient's quality of life. DLQI and HDSS questionnaires and minor's iodine starch test were administered to all patients before and 6 after the treatment with BTX-A, by a physician external to the study.

### 2.1. Statistical Analysis

All data was analyzed using the statistical package for social science (SPSS 17.0) for Windows. Comparisons between groups were done by Mann-Whitney *U* and Friedman-test. The statistical significance was determined at the level of *P* value 0.05.

## 3. Results

The median age of the patients was 29 years and the percentage of female came to 56.3% (67 patients).

Quality of life showed a significant improvement after treatment with BTX-A (Table 1). The total DLQI score resulted significantly lower than the basal value (*P* < 0.0001). The severity of hyperhidrosis significantly decreased after the treatment (median decrease of 3; *P* < 0.0001).

The total score of DLQI and the severity of hyperhidrosis by sex are reported in Table 2 (pretreatment and posttreatment). There was no statistical difference between the pretreatment DLQI scores (*P* = 0.49) and posttreatment severity of hyperhidrosis (*P* = 0.08) of male and female patients. On the other hand, there was notable difference between the posttreatment DLQI scores (*P* = 0.02) and pretreatment severity of hyperhidrosis (*P* = 0.001) by gender.

## 4. Discussion

Hyperhidrosis is a disease that provokes functional impairment and may decrease the quality of life. Patients report lower satisfaction and increased perceived limitations at work because of their condition. They often fail to accomplish their work, they change their work habits most of the time, and they cannot get satisfaction by performing work activities. Furthermore many consider that their productivity at work is affected because of hyperhidrosis. Patients often spend much time and effort managing their disease. They change their clothing many times per day. They also feel emotionally impaired because of their hyperhidrosis, lacking self-confidence and feeling unhappy, depressed, anxious, while also suffering from social phobia [12] and restricted with many daily activities. Patients often report problems in their personal relationships because they feel limited in developing personal relationships, both in their own family and friends and engaging in sexual activities. Their social life is affected because they feel limited in being in public places, meeting people, especially for the first time, participating in sports and shaking hands [13, 14]. In addition, excessive sweating can lead to odor production and in severe cases can result in skin maceration and eventually in secondary infection, such as tinea pedis, viral warts, and dermatitis [2].

The effectiveness and ease of use of BTX-A for the treatment of primary focal hyperhidrosis have been shown in a number of studies. Naumann et al. studied patients suffering from axillary hyperhidrosis, who were treated with BTX-A 50 U per axilla and they were followedup for 16 weeks. The patients treated with BTX-A showed a high and rapid response (from 81.8% to 95%), compared with the placebo group (from 20.5% to 37.2%) [2, 4]. Lowe et al. also showed a long-term efficacy of BTX-A in therapy of axillary hyperhidrosis and the mean duration of the effect was 6 months [2, 5]. Bodokh and Branger showed an improvement in 75% of the patients treated with BTX-A for palmar hyperhidrosis [6, 7, 10]. In another study Naumann et al. demonstrated that patients treated with BTX-A were satisfied in 93% at week 16 posttreatment [14].

In the present study the majority of patients had HDSS score of 3 (26.1%) or 4 (63.9%) and the first line therapy is BTX-A [11]. After the treatment the HDSS score of the majority of the patients is 1 (64.7%) and 2 (33.6%). There was statistically significant difference in the pretreatment severity of hyperhidrosis between male and female but not posttreatment. Campanati et al. in a study of 79 patients suffering from axillary and palmar hyperhidrosis showed that HDSS decreased from a pretreatment value of 4 to a posttreatment value of 0 [3]. Solish et al. studied 146 patients with primary axillary hyperhidrosis and the 64% of patients scored 4 on the HDSS pre-treatment, while at week 4 after treatment 85% of patients scored 1 or 2 on the HDSS and 90% of patients at week 12 [11].

The treatment with BTX-A has radically changed the management of patients with hyperhidrosis [10]. In the present study modified DLQI score before treatment was high (median = 20), which indicates that hyperhidrosis influences significantly the daily functions of the patients. Quality of life improves significantly after BTX-A and median DLQI score is 3. There was no statistically substantial difference between the pretreatment DLQI scores, but there was notable difference between the posttreatment DLQI scores by gender. There are several studies that demonstrate improvement in their quality of life after BTX-A injections. Swartling et al. reported a 76% improvement in DLQI score after treatment with BTX-A and DLQI was reduced from 9.9 to 2.4 for patients who had not relapsed 5 months posttreatment [15]. Tan and Solish showed that DLQI score was reduced significantly after treatment of axillary and palmar hyperhidrosis with BTX-A. For axillary hyperhidrosis DLQI score before treatment was 18 and after treatment it was 4. For palmar hyperhidrosis DLQI score before and after treatment was 17 and 9, respectively. The patients reported an improvement in their self-confidence and changes in their daily habits. The duration of the effect was on average 4 to 7 months [9]. Campanati et al. reported that quality of life improved after treatment and the median DLQI before and after treatment was 13 and 1, respectively. Also quality of life before and after treatment was independent of the involved areas [10]. In another study of 79 patients, Campanati et al. reported that DLQI score before and after therapy was 14 and 1, respectively. Their study also showed that relapse-free survival was shorter in patients suffering from palmar hyperhidrosis and patients with disease duration of 20 years or more [3]. Naumann and Lowe in a study of 320 patients suffering from axillary hyperhidrosis demonstrated that patients who were treated with BTX-A noticed significant improvement in emotional status, in their daily life, in their social activities, and in their productivity at work [4]. Solish et al. also showed that mean DLQI score was reduced from 10.6 before treatment to 1.7 at week 12 after treatment. The majority of patients reported DLQI score of 0 at week 4 and at week 12, which shows that they had no disease related quality of life impairment. Patients also reported more satisfaction and less limitations at work, less effort managing their hyperhidrosis, and improvement in their emotional impairment and in their ability to function in personal relationships and social situations after treatment with BTX-A [13].

The present study included a large number of patients suffering from primary palmar, plantar, and axillary hyperhidrosis. We also demonstrated significant improvement in quality of life of patients, which is in accordance with other studies [3, 9, 10, 12, 13, 15]. Furthermore, it is important for BTX-A treatment because the effective treatment depends on patient's perceived impairment and quality of life.

## Figures and Tables

**Table 1:** Comparison of DLQI and the severity of hyperhidrosis before and after treatment with BTX-A in patients with hyperhidrosis.

	Before treatment with BTX-A	After treatment with BTX-A	*P* value
Total score of DLQI (median (25th–75th percentiles))*	20 (17, 23)	3 (2, 4)	0.0001
The severity of hyperhidrosis (*n* (%))*			
1	1 (0.8%)	77 (64.7%)	0.0001
2	11 (9.2%)	40 (33.6%)	
3	31 (26.1%)	2 (1.7%)	
4	76 (63.9%)	—	

*Friedman test.

**Table 2:** Comparison of DLQI and the severity of hyperhidrosis across sex, before and after treatment with BTX-A.

	Male	Female	*P* value
Total score of DLQI before treatment with BTX-A (median (25th–75th percentiles))*	18.8 (17.7, 19.9)	21 (19.9, 22.1)	0.015
Total score of DLQI after treatment with BTX-A (mean (95% CI))*	3.2 (2.9, 3.6)	3.1 (2.7, 3.5)	0.489
The severity of hyperhidrosis before treatment with BTX-A (*n* (%))*			
1	1 (1.9%)	—	0.001
2	9 (17.3%)	2 (3%)	
3	17 (32.7%)	14 (20.9%)	
4	25 (48.1%)	51 (76.1%)	
The severity of hyperhidrosis after treatment with BTX-A (*n* (%))*			
1	38 (73.1%)	39 (58.2%)	0.079
2	14 (26.9%)	26 (38.3%)	
3	—	2 (3%)	

*Mann-Whitney test.
